# Ankylosing Spondylitis and Posture Control: The Role of Visual Input

**DOI:** 10.1155/2015/948674

**Published:** 2015-03-03

**Authors:** Alessandro Marco De Nunzio, Salvatore Iervolino, Carmela Zincarelli, Luisa Di Gioia, Giuseppe Rengo, Vincenzo Multari, Rosario Peluso, Matteo Nicola Dario Di Minno, Nicola Pappone

**Affiliations:** ^1^Department of Translational Research and Knowledge Management, Otto Bock HealthCare GmbH, 37115 Duderstadt, Germany; ^2^Rheumatology and Rehabilitation ResearchUnit, “Salvatore Maugeri” Foundation I.R.C.C.S., Scientific Institute of Telese Terme, Via Bagni Vecchi 1, 82037 Telese Terme, Italy; ^3^Department of Cardiology, “Salvatore Maugeri” Foundation I.R.C.C.S., Scientific Institute of Telese Terme, Via Bagni Vecchi 1, 82037 Telese Terme, Italy; ^4^Rehabilitation Research Unit, “Salvatore Maugeri” Foundation I.R.C.C.S., Scientific Institute of Cassano delle Murge, Via Mercadante 2, 70020 Cassano delle Murge, Italy; ^5^Department of Clinical Medicine and Surgery, University Federico II, Via Sergio Pansini 5, 80131 Naples, Italy

## Abstract

*Objectives*. To assess the motor control during quiet stance in patients with established ankylosing spondylitis (AS) and to evaluate the effect of visual input on the maintenance of a quiet posture. *Methods*. 12 male AS patients (mean age 50.1 ± 13.2 years) and 12 matched healthy subjects performed 2 sessions of 3 trials in quiet stance, with eyes open (EO) and with eyes closed (EC) on a baropodometric platform. The oscillation of the centre of feet pressure (CoP) was acquired. Indices of stability and balance control were assessed by the sway path (SP) of the CoP, the frequency bandwidth (FB1) that includes the 80% of the area under the amplitude spectrum, the mean amplitude of the peaks (MP) of the sway density curve (SDC), and the mean distance (MD) between 2 peaks of the SDC. *Results*. In severe AS patients, the MD between two peaks of the SDC and the SP of the center of feet pressure were significantly higher than controls during both EO and EC conditions. The MP was significantly reduced just on EC. *Conclusions*. Ankylosing spondylitis exerts negative effect on postural stability, not compensable by visual inputs. Our findings may be useful in the rehabilitative management of the increased risk of falling in AS.

## 1. Introduction

Ankylosing spondylitis (AS) is a chronic inflammatory joint disease, enclosed in the group of spondyloarthritis, most commonly affecting the axial skeleton, usually associated with the presence of HLA-B27 [1]. Sacroiliitis and spine stiffness, due to consolidation of the articulating surfaces and inflammation, represent major clinical features of the disease [2]. Usually postural changes may be observed in early disease, becoming more marked over time. With few exceptions, AS also leads to a rigid thoracolumbar kyphotic deformity. Consequently, patients standing in a stooped position exhibit difficulties in looking up [3] and in performing daily living activities. Moreover, in this clinical setting, poor posture may induce impairment of balance and higher risk of falls [4]. On one hand, the pathophysiological mechanism of disability in AS represents a very intriguing issue to be addressed since it provides new therapeutic opportunities in the management of these patients. In our knowledge, in literature little is reported about balance impairment in AS [5, 6]. Some reports showed that AS patients exhibit a poorer balance as compared to normal subjects [4] and a reduced ability to balance themselves after position changes [7]. Postural balance is a complex function involving many neuromuscular processes [8, 9]. It is controlled by sensory input, central processing, and neuromuscular responses, including the vestibular, visual, and proprioceptive systems. Balance control is essential in all postural conditions, both static and dynamic, requiring integrity of neuromuscular system and an adequate muscle strength. Moreover, previous studies demonstrated the key role of visual inputs in achieving a steady posture control in normal subjects [10, 11], especially in the elderly [12, 13] and in subjects affected by neuromuscular diseases [14–17]. Furthermore, rehabilitation programs based on the recovery of visuomotor integration have been found useful in improving equilibrium [18–21]. In this regard, due to the severity of the disease, AS patients could display an impaired neuromuscular control system and balance ability, especially in absence of the visual input inflow. Therefore, they may exhibit a real dependence on the visual inputs to keep a correct posture control in static condition. Accordingly, our study is aimed to assess the standing upright control and to evaluate how the visual inflow affects the achievement of posture balance in AS patients. The quantitative assessment of posture control impairment both in open and closed eyes condition could be useful in understanding the alterations of the complex system that integrates this sensory information in AS patients.

## 2. Methods

### 2.1. Patients

From December 2011 to May 2012, 12 consecutive male subjects (mean age 50.1 ± 13.2 years, mean disease duration 20.1 ± 13.2 years) were diagnosed with AS according to the modified New York criteria [22] referring to the Rheumatology and Rehabilitation Research Unit of the “Salvatore Maugeri” Foundation (Telese Terme, Italy), and 12 matched healthy controls (12 males, mean age 43.5 ± 4.7 years) entered the study. All patients were treated with standard dosages of an anti-TNF-*α* agent (Infliximab, 5–8 mg/kg each 6–8 weeks) for at least 12 months [23]. In order to avoid any modifications of motor-control performance, AS subjects did not receive any rehabilitative treatment during the study. The healthy group consisted of 12 subjects enrolled among hospital personnel and matched AS patients as to demographical and anthropometric features. All patients were classified as affected by “severe AS” according to the criteria by Murray et al. [5]. In order to minimize the effect of the morning stiffness, all clinical, functional, and instrumental examinations of posture control were carried out at least 3 hours after awakening. Patients underwent a morphological examination of the spine and an instrumental assessment of posture by specific tools. Moreover, a complete clinical evaluation was performed by a trained staff. Exclusion criteria were age >70 years, known balance or vestibular disorders, concomitant severe cardiovascular, neurological, or psychiatric disease, diabetes, and severe visual or auditory impairments (reduced visual acuity was accepted if adequately corrected). Patients with attested orthopedic diseases affecting spine (fractures, spinal disc herniation, spinal surgery, etc.) or lower limbs (prosthesis) were also excluded. Patients and controls were not treated with drugs affecting the central or peripheral nervous system. The study was approved by the local ethics committee and informed consent was obtained from patients and controls.

### 2.2. Clinical Assessment

The assessment of AS was obtained through appropriate physical examination. Functional status and measures of disease activity were obtained by established criteria. The Bath Ankylosing Spondylitis Functional Index (BASFI) [24] was performed to determine the degree of function limitation. Disease activity was measured by the Bath Ankylosing Spondylitis Disease Activity Index (BASDAI) [25]. The five clinical measurements (chest expansion, modified Schober' test, occiput to wall, cervical rotation, and lateral spinal flexion) providing the Bath AS Metrology Index (BASMI) [26] were also accounted.

### 2.3. Instrumental Assessment of Posture, Task, and Procedures

Posture control in upright stance was quantized by a baropodometric platform (FDM-S, Zebris, Germany) [27, 28]. This quantitative posturography measures the forces exerted on the ground during quiet stance obtaining an index named centre of feet pressure (CoP). The latter represents the resulting body sway and the point location of forces used to keep the body mass center projection within the platform [29]. The CoP trajectory is a key output of a complex system [30, 31] that integrates several sensory inputs (visual, somatosensory, vestibular), providing information on nervous and musculoskeletal systems' ability to generate an adequate postural balance [32]. Accordingly, posture control disorders can be detected by changes of CoP spatiotemporal features [33].

Patient and controls were naïve to these instrumental evaluations. The participants were asked to stand quiet on the baropodometric platform as still as possible in their usual posture with arms relaxed on body sides in two visual conditions: (1) eyes open (EO), looking at a visual target adjusted for eyes' height at a 40 cm-distance, and (2) eyes closed (EC). They stood barefoot on the platform with feet spaced 17 cm apart [34]. The room was illuminated with diffuse light and background noise was very low. Trials in which sharp directional sounds unexpectedly occurred were eliminated. Aforementioned precautions were required to simulate “natural” position during evaluations. Subjects and controls were instructed to keep the gaze fixed on a cross target embedded by two vertical lines [35] and stand still for at least 50 seconds. Each subject performed a series of 6 consecutive trials [36], 3 in EO and 3 in EC condition. The visual conditions were randomly assigned and a 1-minute rest was given every two trials to avoid participant discomfort or pain in the soles. Feet position was marked on the platform to assure consistency across trials.

### 2.4. Detection and Analysis of the Center of Feet Pressure (CoP) by Baropodometric Platform

The acquisition time was 50 seconds. We discarded the first and last 10 seconds signals to avoid any transient periods and to analyze exclusively a stationary posture. The CoP displacement was computed off-line calculating 4 parameters (2 global and 2 structural) as recommended in clinical practice [37]. The 2 global posturographic parameters express the “extent” of the CoP oscillations in the time and frequency domains, while structural posturographic parameters examine the CoP sway patterns related to the motor control activity (posturographic motor commands). For each trial in EO and EC conditions, we computed the 2 following “global” parameters: (i) the sway path (SP) of the CoP, integrating the instantaneous velocity of the CoP over the total acquisition time. SP measures the mean velocity of CoP oscillations; therefore, its increasing addresses a reduced posture stability. (ii) The frequency bandwidth (FB1) includes the 80% of the area under the amplitude spectrum [37], for both anteroposterior (A-P) and mediolateral (M-L) directions. FB1 measures the amount of quick transient CoP displacements, separately for the frontal and sagittal anatomic planes. The FB1 increasing represents an enhancement in the effectiveness of the posturographic motor commands which account for a faster control of CoP oscillations. The 2 “structural” parameters were calculated from the sway density curve (SDC). The SDC is constructed by counting the number of consecutive points of the CoP trajectory that, for each time instant, fall inside a test circle of a radius of 2.5 mm [37]. Therefore, SDC presents a regular alternation of peaks and valley. Peaks correspond to time instants in which the CoP control is relatively stable, while valleys correspond to instants in which the CoP control rapidly shifts from one stable point to the next one. Following are the 2 calculated structural parameters: (iii) the mean amplitude of the peaks (MP) of the sway density curve. MP is directly related to the degree of stability obtained by posturographic motor commands; therefore, MP increasing represents an increased quantity of CoP trajectory stable points; (iv) the mean distance (MD) between two consecutive peaks of the sway density curve. [37]. Its reduction shows a faster and more efficacy release of posturographic motor commands.

### 2.5. Statistical Analysis

Continuous data were expressed as mean ± standard deviation; categorical variables were expressed as %. For every group we executed a paired Student's* t*-test between EO and EC data to evaluate significant differences between visual conditions, while for all the other statistical analysis we adopted an unpaired Student's* t*-test. The Pearson's *r* was used to perform correlation between continuous variables. The chi-square test was performed to compare categorical data. When the minimum expected value was <5, Fisher's exact test was used. All the results are given as two-tailed values with statistical significance for *P* values <0.05. For each subject and control, we calculated the mean value of the measured variables. For all statistics, the significance level was *P* < 0.05. Average values ± standard deviation (SD) over all trials for all subjects and measured variables were also computed.

## 3. Results

The demographic, anthropometric, and clinical features of study population are shown in Table 1. There were no significant differences between the groups according to age, gender, height, weight, or body mass index (BMI). Table 1 also reports the results of physical evaluations performed for each patient before instrumental posture assessment.

**Figure 1:**
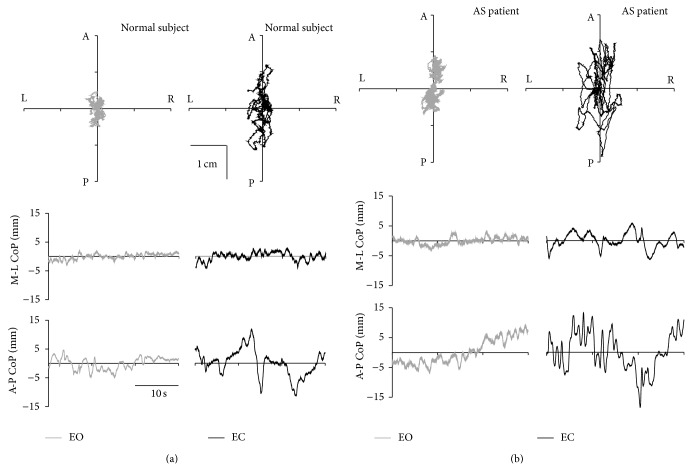
Statokinesigrams (upper 2D plots of the planar oscillation of the CoP) and stabilograms (lower traces plotted against time) of a normal subject (left half) and an AS patient (right half) plotted during eyes open (EO, gray lines) and eyes closed (EC, black lines). The stabilograms are the projection of the statokinesigram along the mediolateral (M-L, 2nd row plots) and anteroposterior (A-P, 3rd row of plots) directions of the subject. A = anterior, P = posterior, L = left, R = right.

Figure 1 shows representative plots of CoP oscillation obtained from a healthy control and an AS patient during EO and EC conditions. For both groups, the support postural base is larger along anteroposterior (A-P) direction than mediolateral (M-L) direction. Accordingly, the extent of CoP oscillations is more marked along A-P direction than M-L direction [38]. The figure also shows increased oscillation amplitude for both groups switching from EO to EC especially along A-P direction. In particular, the figure qualitatively reports the motor postural control impairment in AS. Of interest, we found differences in terms of CoP peak to peak amplitude, oscillation frequency, and the area covered by the CoP oscillations between controls and AS patients during both visual conditions.

### 3.1. Balance Behavior between Eyes-Open (EO) and Eyes-Closed (EC) Trials

**Figure 2:**
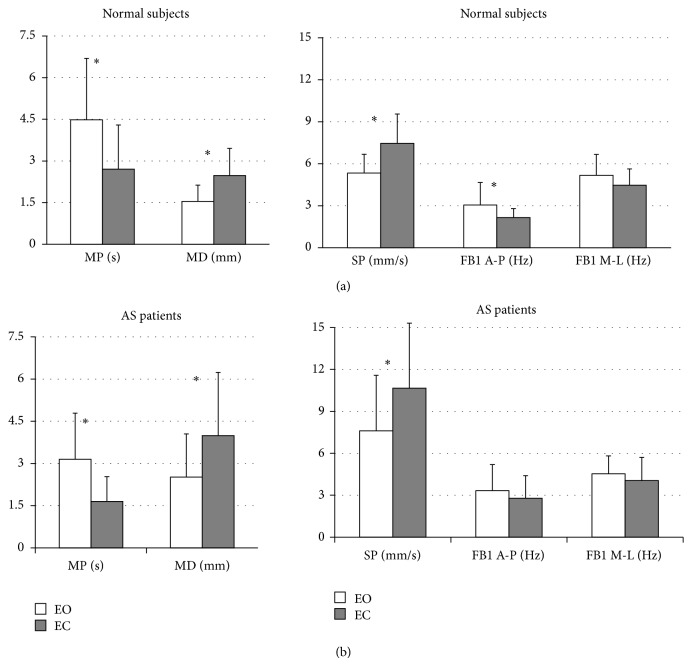
Structural parameters (MP and MD, left side) and global parameters (SP, FB1 along A-P and M-L directions, right side), for normal subjects (upper panels) and AS patients (lower panels) during eyes open (EO, white columns) and eyes closed (EC, black columns). Data are expressed as mean ± standard deviation. The asterisks represent significant difference (*P* < 0.05).

**Figure 3:**
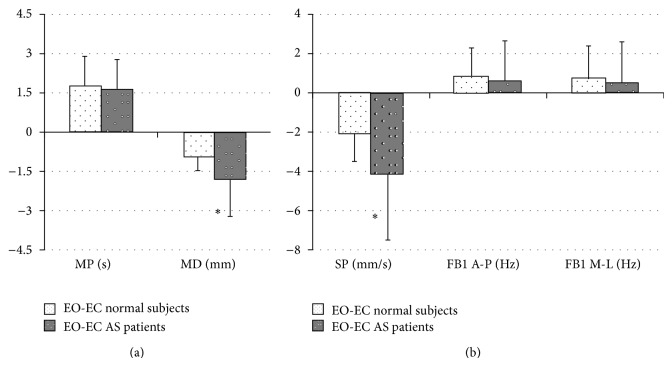
Mean ± standard deviation of the differences between EO and EC values of the structural parameters (MP and MD, left side) and global parameters (SP, FB1 along A-P and M-L directions, right side), for normal subjects (white columns with black dots) and AS patients (black columns with white dots). The asterisks represent significant difference (*P* < 0.05).

As shown in Figure 2 the mean values of CoP parameters were significantly different switching from EC to EO condition. In details, MP, MD, and SP values were found to be significantly different for both control and AS groups (*P* < 0.005), while FB1 along AP direction a significant difference was found only in the control group (*P* < 0.005). These findings reveal worsening of upright stance control from EO to EC condition. Therefore, in order to highlight the differences between the groups, we also evaluated the delta (Δ) between EO and EC for each parameter (Figure 3). We found a statistically significant difference only for Δ-MD and Δ-SP values between groups (*P* < 0.05, Figure 3). Moreover, the figure shows no difference in CoP oscillation frequency (Δ-FB1) and in stabilizing duration time (Δ-MP) between the groups.

**Figure 4:**
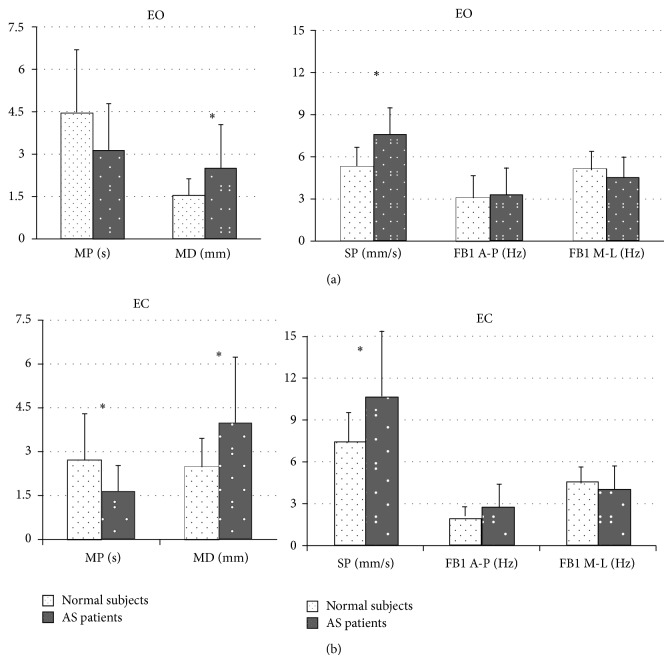
Structural parameters (MP and MD, left side) and global parameters (SP, FB1 along A-P and M-L directions, right side), for normal subjects (white columns with black dots) and AS patients (black columns with white dots). Data are expressed as mean ± standard deviation. The asterisks represent significant difference (*P* < 0.05).

In Figure 4 we report the comparison between subjects and controls according to the two visual conditions. MD and SP values were found to be significantly different between the groups (*P* < 0.05), while MP was found to be different only in EC condition (*P* < 0.05).

Finally, Pearson's correlation, performed to investigate the relationship between posture control indices and AS clinical outcomes, showed that BASMI was significantly related to MP values (*r* = −0.57; *P* = 0.042), while BASFI or BASDAI did not show a significant correlation with one of the four posturographic parameters assessed (*P* > 0.05).

## 4. Discussion

To generate goal-directed movements, such as reaching with the arm at stationary or moving objects, the brain must integrate external inputs (e.g., visual, auditory) relative to target position with intrinsic signals (proprioceptive, vestibular, motor) related to body, head, and eye positions.

The posterior parietal cortex, which is part of the visual dorsal stream, is “reciprocally” connected with motor areas of the frontal lobe representing an important sensorimotor interface for movements and posture control. To circumvent sensory feedback delays, current motor control theories postulate the existence of “forward” models, combining sensory inputs with motor commands [9]. On one hand, given the posture deterioration in AS, our study represents the first assessment of the posture stability in these patients with and without the support of visual inputs, showing that the latter is not able to effectively improve posture stability in the setting. As qualitatively and quantitatively shown in Figures 1 and 2, the availability of visual information affects postural control in both groups. Accordingly, Figure 1 shows that in healthy and AS subjects during EC, except for FB1 along M-L direction, all posturographic indices were significantly different as compared to EO, evidencing a worse stability in that condition. On the other hand, only in AS patients we found no difference in FB1 along A-P direction during EO versus EC. These findings, together with the significant increase in SP mean during EC, suggest that in normal subjects the control of equilibrium during EC was achieved with greater oscillations and lower velocity along A-P direction. In AS an opposite effect was found. In details, without the support of visual input, AS patients increase amplitude and velocity of CoP oscillation obtaining a still high frequency in CoP displacement. This finding is supported by the significant difference in the SP index, between the study groups (Figure 3). Moreover, the significant difference between the groups of the Δ mean SP (calculated as the difference between EO and EC) confirmed the higher velocity of the CoP oscillation along the anteroposterior axis for AS patients when their balance was not supported by visual input. This finding shows an excessive reliance on visual information in AS patients and a consecutive increased posture instability during EC as compared to normal subjects. Of interest, Figure 3 shows another difference between the groups. In particular, considering both MD and MP results, we may suggest that AS patients in EC condition were able to perform larger but still quicker posturographic motor commands as compared to normal subjects. This statement is further confirmed by results reported in Figure 4, showing only in EC condition a significant difference for both MP and MD values in the groups. Since AS patients have no central nervous system diseases, such modification of motor control should not derive from the deterioration of the visual sensory pathway but might be a consequence of a likely impairment of the motor controller, that is, the inability to generate quick and precise motor commands. This is evidenced by a higher amplitude of CoP oscillations (reported as higher SP values, for both EO and EC, Figure 4) and by higher MD values, especially in EC (Figure 4). Moreover, the significant differences in MD mean values, between the groups for both visual conditions (Figure 4), as well as for the Δ mean MD (difference between EO and EC, Figure 3), could be explained by an alteration of the intrinsic feedback due to the mechanical properties of the lower limb muscles, modulated by the segmental reflexes. As shown by their larger support base (Figure 1) during EC, in AS patients the impaired ability of adopting anticipatory muscle activations in controlling the upright quiet stance posture could lead to an increased risk of falling. The impaired posture control has been already correlated with an increased risk of falling in elderly [39–41] and neuromuscular disease patients [42–44]. Falling and related problems, that is, fear of falling and daily life activity restriction, are known to contribute to consistently reducing quality of life [45]. Moreover, the correlation analysis suggests a strong link between the degree of posture stabilization, measured by MP parameter in EO and EC condition and spinal mobility assessment (BASMI). Accordingly, by BASMI evaluation, we could eventually predict the status of motor controller degradation. Our findings are in line with previous studies, which showed that AS exhibits negative effect on postural stability, [5, 6] even by different instrumental tools. These effects are more evident in the later stages of the disease [5].

In conclusion, our study, showing that the posture control in AS is not effectively improved by visual input, confirms the initial hypothesis about the existence of “forward” models, combining sensory inputs with motor commands. In particular, according to our finding, we could speculate that a chronic postural imbalance, linked to the inflammatory spine disease, may affect the central sensory feedback. As a consequence, these patients show a reduced the ability of visual input to improve body stability. Accordingly, these results may allow applying new rehabilitation methods based on increasing proprioceptive inflow integration with rehabilitating exercises executed without visual information support. Finally, this study points out a further important issue on the correlation between severe AS and risk of falling. It highlights the importance of developing advanced rehabilitation programs aimed at reducing the CoP amplitude oscillations especially without visual input. These programs could increase the safety margin of the posture motor commands' intervention. Indeed, monitoring patients' balance may be of interest to develop new rehabilitation and protection methods aimed to increase postural stability and protect the elderly and severely affected patients from falling and its associated sequelae.

## Figures and Tables

**Table 1:** Demographic, anthropometric, and clinical features of study population.

	AS subjects	Control subjects	*P*
Height (cm) mean ± sd	172.4 ± 9.6	166.3 ± 9.9	0.14
Weight (Kg) mean ± sd	84.4 ± 20.5	72.7 ± 16.8	0.16
BMI (Kg/m^2^) mean ± sd	30.3 ± 6.7	26.2 ± 5.4	0.12
Age (years) mean ± sd	50.1 ± 13.2	43.5 ± 4.7	0.11
Disease duration (years) mean ± sd	20.1 ± 13.2	/	
BASFI mean ± sd	41.6 ± 22.5	/	
BASDAI mean ± sd	45.9 ± 29.9	/	
BASMI mean ± sd	6.6 ± 1.5	/	
Male gender	100%	100%	1.000

## List of Abbreviations

A-P: Anteroposterior
AS: Ankylosing spondylitis
BASDAI: Bath ankylosing spondylitis disease activity index
BASFI: Bath ankylosing spondylitis functional index
BASMI: Bath ankylosing spondylitis metrology index
CoP: Centre of foot pressure
EC: Eyes closed
EO: Eyes open
FB1: Frequency bandwidth that includes the 80% of the area under the amplitude spectrum of the centre of foot pressure oscillations
MD: Mean distance between one peak and another of the sway density curve of the centre of foot pressure
M-L: Mediolateral
MP: Mean amplitude of the peaks of the sway density curve of the centre of foot pressure
SDC: Sway density curve of the centre of foot pressure
SP: Sway path of the centre of foot pressure.
